# Intrapleural hypotonic cisplatin treatment for malignant pleural effusion in 80 patients with non-small-cell lung cancer: a multi-institutional phase II trial

**DOI:** 10.1038/sj.bjc.6603319

**Published:** 2006-08-29

**Authors:** T Seto, S Ushijima, H Yamamoto, K Ito, J Araki, Y Inoue, H Semba, Y Ichinose, Kyushu Yamaguchi Thoracic Oncology Group

**Affiliations:** 1 Department of Thoracic Oncology, National Kyushu Cancer Center, 3-1-1, Notame, Minami-ku, Fukuoka 811-1395, Japan; 2 Department of Respiratory Medicine, Kumamoto Central Hospital, Kumamoto, Japan; 3 Department of Respiratory Medicine, Asou Iizuka Hospital, Fukuoka, Japan; 4 Department of Respiratory Medicine, Shin Beppu Hospital, Oita, Japan; 5 Department of Respiratory Medicine, Yamaguchi Central Hospital, Yamaguchi, Japan; 6 Department of Respiratory Medicine, Isahaya Insurance General Hospital, Nagasaki, Japan; 7 Division of Respiratory Diseases, Kumamoto Regional Medical Center, Kumamoto, Japan

**Keywords:** non-small-cell lung cancer, malignant pleural effusion, intrapleural chemotherapy, management of malignant pleural effusion, hypotonic cisplatin treatment

## Abstract

To assess the effect and toxicity of hypotonic cisplatin treatment (HPT) consisting of the intrapleural administration of cisplatin in distilled water for malignant pleural effusion in patients with non-small-cell lung cancer (NSCLC). Non-small-cell lung cancer patients with cytologically proven and previously untreated malignant pleural effusion were enrolled into this study. Firstly, the lung was fully re-expanded by a tube thoracostomy, and then 25 mg cisplatin in 500 ml of distilled water was instilled through a chest tube and then the tube was clamped. After 1 h, the tube was declamped and allowed to drain. The chest tube was removed when the pleural effusion volume decreased to 200 ml or less per day. A complete response (CR) was considered to occur when the pleural effusion disappeared. A partial response (PR) was determined to occur when the volume of pleural effusion remained under ¼ of hemithorax. The response at 4 weeks was evaluated by an extramural review. Out of 84 patients enrolled from February 1998 to August 2002, 80 patients were eligible and analysed in the present study. The toxicity of HPT was acceptable. Neither a haematological toxicity of any grade nor grade 4 nonhaematological toxicity was observed. Grade 3 nonhaematological toxicities were observed, including nausea (4%), vomiting (3%), pyothorax (1%) and dyspnoea (1%). The median time of drainage from HTP was 4 days. Twenty-seven (34%) and 39 (49%) patients achieved CR and PR, respectively, for an overall response rate of 83% (95% confidence interval, 74–91%). The median duration of the response was 206 days. The median survival time of all patients was 239 days. Hypotonic cisplatin treatment for malignant pleural effusion of NSCLC is therefore considered to be feasible and effective. A phase III study of HPT is thus warranted.

Malignant pleural effusion is considered to be the first clinical manifestation of malignancy as well as the first sign of recurrent cancer. Among the various kinds of malignancies, lung cancer is the leading cause of malignant effusion (Chernow and Sahn, 1977; Shan, 1987). The standard treatment for a patient with a symptomatic malignant pleural effusion due to non-small-cell lung cancer (NSCLC), whose life expectancy is not too short, is considered to be a tube thoracotomy with subsequent pleurodesis (Ruckdeschel *et al*, 1991; Walker-Renard *et al*, 1994).

Ichinose *et al* (1997) reported that intraoperative intrapleural treatment using hypotonic cisplatin solution (cisplatin solution diluted by distilled water) effectively controlled malignant pleural effusion and/or pleural dissemination found at thoracotomy in NSCLC patients. According to their experimental data, hypotonic cisplatin solution demonstrated a significantly greater antitumour activity than either isotonic cisplatin or distilled water alone (Ichinose *et al*, 1993).

Ushijima *et al* (1997) applied this hypotonic cisplatin treatment (HPT) consisting of the intrapleural administration of cisplatin in distilled water after tube thoracostomy to treat malignant pleural effusion due to NSCLC and gastric cancer, and reported successful results in several patients. A multi-institutional phase II trial was thus conducted to assess the effect and toxicity of HPT for malignant pleural effusion due to NSCLC.

## PATIENTS AND METHODS

### Patient eligibility

The patients were eligible for this phase II trial if they had cytologically proven and previously untreated malignant pleural effusion of NSCLC, which had either not yet been treated or had been treated 4 weeks or more before enrolment. All participants were required to be under 80 and 80 year of age, with a leucocyte count of ⩽4000 *μ*l^−1^, a platelet count of >100 000 *μ*l^−1^, a serum bilirubin level ⩽1.5 mg dl^−1^, a normal creatinine level and serum glutamic oxaloacetic transaminase/glutamic pyruvic transaminase levels of no more than twice the upper limit of normal. The patients were also required to have a sufficient re-expansion of the lung after chest tube drainage and an Eastern Cooperative Oncology Group (ECOG) performance status (PS) of 0, 1 or 2 after drainage. The extent of re-expansion was re-assessed by an extramural review committee. Systemic chemotherapy or radiotherapy to the lung, mediastinum and pleura was not given for 4 weeks after HPT. This trial was approved by the institutional review boards of each participating institution, and written informed consent was obtained from all patients.

### Treatment methods

The lung was fully expanded by a thoracostomy using a chest tube with a double lumen, and then the patients were enrolled into the trial via facsimile by the administration office of the Kyushu Yamaguchi Thoracic Oncology Group in the National Kyushu Cancer Center. First, premedications, intramuscular injection of 15 mg of pentazocine and intrapleural administration of 10 ml of 1% lidocaine were performed. Thereafter, 25 mg of cisplatin in 500 ml of distilled water was instilled through a chest tube. The hypotonic cisplatin solution was prepared as follows: 50 ml cisplatin solution containing 25 mg cisplatin was injected into the bottle containing distilled water of 500 ml. The chest tube was clamped for 1 h. The patients were then asked to change position (supine and bilateral decubital) from time to time during the treatment regimen, and then the tube was declamped and allowed to drain under a negative pressure of 10–15 cm H_2_O. When the drainage effusion was less than 200 ml a day, the chest tube was removed. Any patient whose drainage effusion continued for over 2 weeks was withdrawn from the trial and was also judged to be a nonresponder.

### Evaluations

Toxicities were evaluated according to ECOG common toxicities criteria (Oken *et al*, 1982).

The response to HPT was evaluated based on the findings of posteroanterior and lateral chest radiographs 4 weeks after HPT. The response criteria used were as follows: a complete response (CR) when no pleural effusion was observed; a partial response (PR) when pleural effusion was observed, but the level of effusion was less than 25% of the long axis of the hemithorax; and no response (NR) when effusion was larger than that defined by PR. A chest radiograph in responding patients was taken at least every month in order to monitor the condition of the controlled pleural effusion. The response and duration of response were determined by an extramural review committee.

A progression of effusion was defined as when pleural effusion of more than 25% of the long axis of the hemithorax was observed or tube drainage was needed. The effusion-progression-free survival time was defined as the time from the enrolment until the progression of effusion or death without a progression of effusion. The overall survival was defined as the time from enrolment until death from any cause.

### Statistical analysis

The primary end point of the study was the overall response rate including CR and PR. Based on the assumption that a response rate of higher than 75% would warrant further investigation of this treatment, and that a rate below 60% would make such an investigation unnecessary, a total sample size of 62 patients was required with an alpha error of 0.05 and a beta error of 0.20, using the mini-max two-stage sequential design by Simon. The first stage of the study required 30 patients, and if at least 18 responses were observed, then a second stage required 32 patients would be conducted. Since ineligible patients would be included, the accrual of at least 70 patients was thus planned.

## RESULTS

### Patient characteristics

Eighty-four patients were enrolled into this trial from February 1998 to August 2002. However, four patients were later judged to be ineligible: two patients had malignant pleural effusion due to either parotid gland cancer or uterus cancer, one patient had bilateral malignant effusion and one patient had a poor PS who had no chest X-ray film in the standing position. As a result, 80 patients were eligible and thus were analysed in the present study. As shown in Table 1, they included 40 men and 40 women with a median age of 67 years. Most patients had an ECOG PS of 0 or 1 and a histology of adenocarcinoma. In addition, 73% of the patients had received no prior therapy. Although the extent of a re-expansion of the lung after tube thoracotomy was judged to be equivocal in four patients at an extramural review committee, those patients were included in the analysis.

### Adverse events

Neither the haematological toxicity of any grade nor of grade 4 nonhaematological toxicity was observed in all 80 eligible patients (Table 2). Grade 3 nonhaematological toxicities were observed, including nausea (4%), vomiting (3%), dyspnoea (1%) and pyothorax (1%).

### Response

Among the 80 patients including one patient whose response was not evaluable due to complications of pyothorax, 27 patients (34%) had CR and 39 patients (49%) achieved PR with an overall response rate of 83% (95% confidence interval 74–91%). There were also 13 patients with NR. Two of these 13 patients were withdrawn from the trial since their effusion could not be controlled within 2 weeks after HPT. The median duration of drainage after HPT in the responding patients was 2 days ranging from 1 to 22 days. The median time of response was 206 days in 66 responding patients ranging 36–949 days.

### Survival

**Figure 1:**
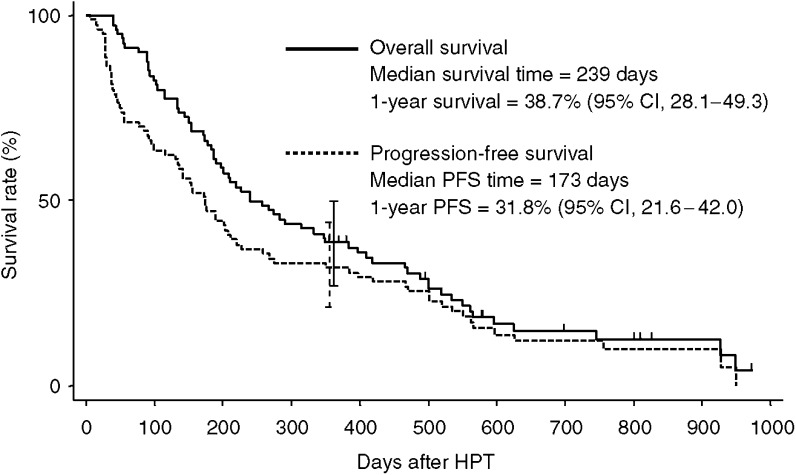
Overall survival and progression-free survival. Each tick mark and bar represents a patient who is alive and the 95% confidence interval of the survival rate, respectively.

The median follow-up period was 1045 days (range, 424–2061 days). The median effusion-progression-free survival time and 1-year effusion-progression-free survival rate of all 80 patients were 173 days and 31.8% (95% confidence interval, 22–42%). The median survival time and the 1-year survival rate of all 80 patients were 239 days and 39% (95% confidence interval, 28–49%) as shown in Figure 1.

## DISCUSSION

The agents administered intrapleurally for the management of malignant pleural effusion were classified as either non-anticancer or anticancer drugs. The non-anticancer drug had a sclerosing agent that produces pleurodesis. The most frequently used agent is talc in the United States (Hausheer and Yarbro, 1985), tetracycline or doxycycline (Putnam *et al*, 1999) in the United Kingdom and OK432, which is prepared from a substrain of *Streptococcus pyrogenes* A3, in Japan (Saka *et al*, 1994). In a majority of cases, talc is administered by pleural pourdage through thoracoscopy using either local anaesthesia or general anaesthesia by the surgical team. Although the response rate to thoracoscopic talc insertion is reported to be over 90%, the requirement of thoracoscopy leads to some limitations in the use of talc. In addition, the frequency of severe chest pain induced by treatment with talc pleurodesis was reported to be 17% (Hartman *et al*, 1993). On the other hand, no such pain was observed in the 80 patients who received this intrapleural HPT for malignant effusion due to NSCLC. The intrapleural administration of the tetracycline or doxycycline was reported to be effective for the control of malignant effusion. However, it has recently become difficult to use tetracycline or doxycycline since these have been withdrawn from the market in 1991 and 1997, respectively. The administration of OK432 in saline solution is easily performed through a chest tube and it is reported to control over 70% of malignant pleural effusion cases. However, its usage is still limited to only Japan.

Although anticancer drugs administered intrapleurally for the management of malignant pleural effusions are expected to have both cytotoxic and sclerosing effects, the mechanism of action for controlling effusion remains unclear. The results of etoposide (Holoye *et al*, 1990), fluorouracil (Suhrland and Weisberger, 1965), mitomycin-C (Luh *et al*, 1992) or doxorubicin (Desai and Figueredo, 1979) investigated in various trials have not proven to be sufficiently attractive and these agents are therefore not presently in use. In a randomised trial comparing intrapleural administration of bleomycin with intrapleural tetracycline, bleomycin has been proven to be superior to tetracycline for the management of malignant pleural effusion (Moffett and Ruckdeschel, 1992). However, bleomycin has not been used extensively due to its high expense. The Lung Cancer Study Group performed a phase II trial of the intrapleural administration of combination chemotherapy using cisplatin and cytarabine in 46 patients, of whom about half had NSCLC (Rusch *et al*, 1991). Tohda *et al* (1999) also performed a similar phase II trial using cisplatin plus etoposide in 70 NSCLC patients. The overall response rate was reported to be 49% in the former trial and 46% in the later trial. As the criteria of the response and proportions of the disease in the subjects vary between the different trials, an accurate comparison of the results is difficult. However, the response rate reported in those intrapleural cisplatin-based chemotherapy trials seems to be, on the whole, inferior to that in the trials using a non-anticancer agent.

In the present phase II trial of the intrapleural administration of hypotonic cisplatin solution in 80 patients with NSCLC, the overall response rate was 83%. The criteria of the response in this trial were similar to those of the cisplatin-based intrapleural chemotherapy trials mentioned above. The main difference between the previously reported intrapleural cisplatin-based chemotherapy and our HPT is the use of isotonic saline in the former and distilled water in the latter for a dilution of cisplatin, which itself is an isotonic solution. Ichinose *et al* (1993) found that hypotonic cisplatin solution whose cisplatin concentration and osmolarity ranged between 5 and 50 *μ*g ml^−1^ and between 2.8 and 28 mOsm l^−1^, respectively, had a significantly stronger antitumour activity than either isotonic cisplatin or distilled water alone in an *in vitro* experiment of short-time exposure ranging from 0.5 to 10 min. In a prospective study in patients whose malignant pleural effusion and/or pleural dissemination were found at thoracotomy, intraoperative intrapleural HPT for 15 min before the closure of thorax prolonged the control of the pleural disease (Ichinose *et al*, 1997). In addition, a randomised phase III trial demonstrated the intraoperative intrapleural HPT in resected NSCLC patients with a positive intrapleural lavage cytology finding to significantly decrease the occurrence of malignant effusion and/or pleural dissemination after operation (Ichinose *et al*, 2002). The cisplatin concentration (50 *μ*g ml^−1^) and hypotonicity (28 mOsm l^−1^) were the same as those in the present trial. The mechanism by which the HPT shows an antitumour effect is considered to be as follows: (1) distilled water itself has a direct cytotoxicity (Ichinose *et al*, 1993), (2) tumour cells exposed to hypotonic cisplatin increase their cellular cisplatin level since the cells become swollen by the hypotonic solution (Ichinose *et al*, 1993) and (3) chloroaqua and diaqua, formed by the hydrolysis of cisplatin in distilled water, are also believed to be active antitumour agents (Rosenberg, 1978; Sherman and Lippard, 1987).

The incidence of the toxicities seen in this study was significantly lower than that in the cisplatin-based chemotherapy trials. Grade 3 non-haematological toxicities were observed, including nausea (4%), vomiting (3%), dyspnoea (1%) and infection (1%) in the present trial, while 90% of the patients had gastrointestinal toxicities including grade 3 and 49 and 9% of the patients experienced haematologic toxicities of grades 1 plus 2 and that of grade 3, respectively, in the trial of the Lung Cancer Study Group. Tohda *et al* (1999) reported a gastrointestinal toxicity of grade 3 in 27% of the patients. The main reasons for a different incidence of toxicities between the two trials and our trial are considered to be due to the differences in intrapleural exposure time of the agent and the administered dosage between trials: (1) the clamping time after the intrapleural administration was only 1 h in our trial compared to either 4 or 72 h in the previous trials and (2) the dose of cisplatin administered was 25 mg body^−1^ once or twice in our trial, while it was 80 and 100 mg m^−2^ in the previous trials. Regardless of the administration of cisplatin with lower dosage as well as the shorter treatment time, the response rate of the HPT, nevertheless, seems to be superior to that of the previously reported chemotherapy trials.

In addition, the overall survival and effusion-progression-free survival curves are closely similar as shown in Figure 1. This observation indicates that malignant pleural effusion was controlled by HPT in most patients.

Although HPT is considered to be a feasible and active treatment for malignant effusion due to NSCLC, further trials are called for. The Japan Clinical Oncology Group (JCOG) conducted a randomised phase II study for malignant pleural effusion due to NSCLC, by comparing bleomycin, OK-432 and cisplatin plus etoposide. We therefore intend to conduct a phase III study to compare HPT with the best arm of the JCOG trial.

## Figures and Tables

**Table 1:** Patient characteristics

Enrolled patients	84
Eligible patients	80
	
*Gender*	
Male	40 (50%)
Female	40 (50%)
	
Age median (range)	67 (35–89)
	
*ECOG performance status*	
0	24 (30%)
1	47 (59%)
2	9 (11%)
	
*Stage*	
IIIB	35 (44%)
IV	45 (56%)
	
*Histological type*	
Adenocarcinoma	77 (96%)
Squamous cell carcinoma	3 (4%)
	
*Prior therapy*	
None	58 (73%)
Surgical resection only	7 (9%)
Chemotherapy or radiotherapy	10 (13%)
Surgical resection plus chemotherapy or radiotherapy	5 (6%)
	
*Re-expansion of lung*	
Sufficient	76 (95%)
Equivocal	4 (5%)

**Table 2:** Nonhaematological toxicities

**Grade**	**1**	**2**	**3**	**4**
Nausea	21	11	3 (4%)	0
Vomiting	7	7	2 (3%)	0
Fever	11	0	0	0
Dyspnoea	5	2	1 (1%)	0
Chest pain	13	5	0	0
Infection	0	0	1 (1%)	0
